# Styloidogenic Internal Carotid Artery Dissection Presenting as Severe New-Onset Headache in a Young Adult

**DOI:** 10.7759/cureus.115130

**Published:** 2026-08-25

**Authors:** Azad Kala Bhushan, John Omomila, Catherine E Harris, Jane Sword

**Affiliations:** 1 Internal Medicine, Royal Devon University Healthcare NHS Foundation Trust, Exeter, GBR; 2 Stroke Medicine, Royal Devon University Healthcare NHS Foundation Trust, Exeter, GBR

**Keywords:** carotid arterial dissection, headache, stroke, stroke in young adults, stylocarotid artery syndrome, vascular eagle syndrome

## Abstract

Cervical artery dissection is a leading cause of stroke in young adults and may present with isolated headache without objective focal neurological deficit. Vascular Eagle syndrome (also known as stylocarotid artery syndrome) is a specific variant of Eagle syndrome, characterised by an elongated styloid process that may mechanically irritate or compress the internal carotid artery, predisposing it to dissection. We report a case of a 31-year-old man who presented with severe new-onset headache and was found on CT angiography to have internal carotid artery dissection in the context of an elongated styloid process consistent with vascular Eagle syndrome. His brain MRI demonstrated no acute infarction. He was managed with antiplatelet therapy and discharged with stroke follow-up and planned interval CT angiography. This case highlights the importance of considering cervical artery dissection in young patients with severe headache even without neurological deficit, the diagnostic value of CT angiography in identifying both the dissection and underlying structural contributors, and the clinical significance of recognising stylocarotid artery syndrome (vascular Eagle syndrome) as a potentially reversible mechanical risk factor triggering this vascular phenomenon.

## Introduction

Cervical artery dissection is an important cause of ischaemic stroke in young adults, accounting for up to 25% of strokes in patients under 50 years of age. It may present with isolated headache or neck pain without focal neurological deficits, making early diagnosis challenging [1]. While many cases are spontaneous or follow minor trauma, structural factors can contribute. Eagle syndrome is a rare condition referring to symptomatic elongation of the styloid process beyond 3 cm and/or stylohyoid ligament calcification, occurring in approximately 4% of the general population [2]. In its vascular form (stylocarotid syndrome), the elongated styloid process may mechanically irritate or compress the internal carotid artery (ICA), predisposing to dissection or thromboembolism [3]. Recognition of this structural risk is clinically important, as management may require antithrombotic therapy and multidisciplinary consideration of styloidectomy or stenting in selected patients to reduce recurrence risk [3].

## Case presentation

A previously healthy 31-year-old right-handed man presented to the emergency department with a seven-day history of worsening headache. The headache began suddenly, was initially intermittent, and progressively increased in severity, reaching 10/10 at its worst. It was described as severe pressure-like pain maximal at the vertex, radiating bilaterally to the temples.

Associated symptoms included intermittent nausea and paraesthesia affecting both upper limbs and both feet. He also described subjective heaviness predominantly affecting the right arm, without weakness on examination. The pain disrupted sleep and occasionally limited exertion such as climbing stairs.

He had no prior history of migraine. There were no visual symptoms, photophobia, fever, neck stiffness, or preceding viral illness. He denied recent trauma, loss of consciousness, and contact sports and did not report consistent provocation with neck rotation. He took no regular medications. Family history included maternal subarachnoid haemorrhage due to ruptured intracranial aneurysm at age 41.

Examination showed normal observations, including blood pressure. Neurological examination revealed no objective focal deficit; cranial nerves, motor, sensory, coordination, and gait were all normal. However, a normal neurological examination would not rule out a carotid dissection, particularly given that his symptoms were acute, ongoing, and significantly debilitating enough for him to seek urgent medical help.

The initial working diagnosis included subarachnoid haemorrhage given the sudden onset and severe nature of the headache, particularly with a family history of aneurysmal rupture. However, the combination of severe headache with intermittent neurological symptoms (paraesthesia and subjective unilateral limb heaviness), even in the absence of objective deficit, raised concern for vascular pathology, including cervical artery dissection. Migraine was considered but deemed less likely given the absence of prior migraine history and the atypical persistent presentation. Other considerations included intracranial aneurysm, cerebral venous sinus thrombosis, and meningitis, though the clinical picture and absence of systemic features made these less likely.

Investigations

**Figure 1:**
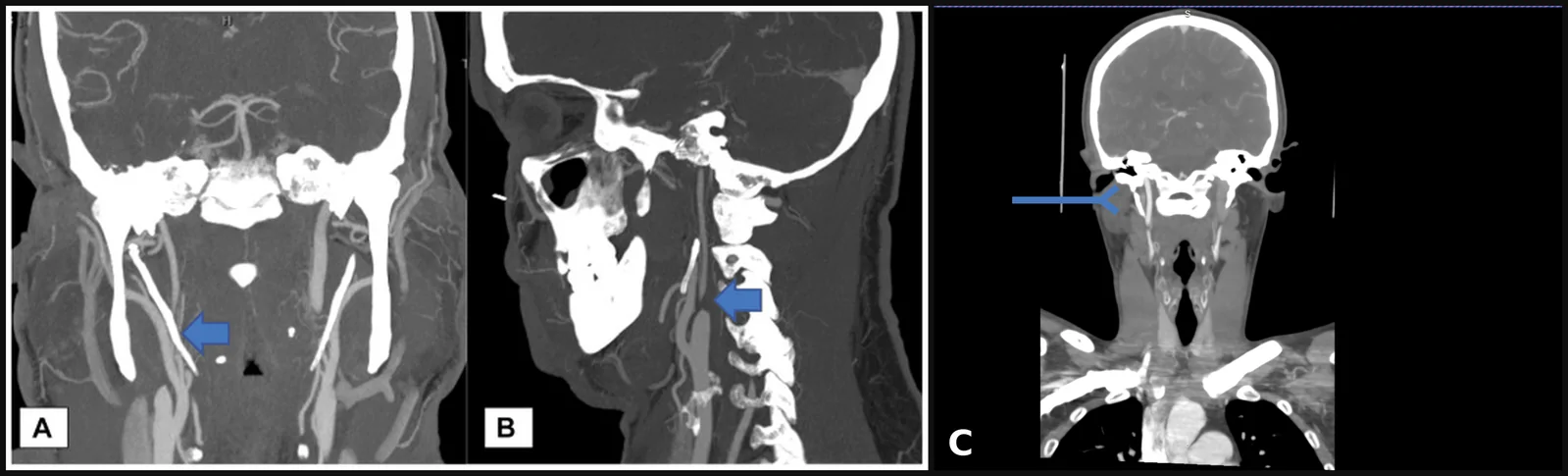
CT angiography of the head and neck demonstrating left internal carotid artery (ICA) dissection in the context of vascular Eagle syndrome (stylocarotid syndrome) CT angiography of the head and neck demonstrating left ICA dissection. (A) Coronal maximum intensity projection (MIP) view showing occlusion of the left ICA from the level of the common carotid bifurcation with progressive luminal tapering (arrow). Note the asymmetry between the patent right ICA and the occluded left side. (B) Sagittal reconstructed view confirming left ICA dissection with luminal irregularity and near-complete occlusion at the carotid foramen (arrow). Re-opacification of the distal left ICA territory was demonstrated via the left posterior communicating artery. (C) Coronal view at the level of the skull base demonstrating the elongated left styloid process in close proximity to the affected ICA segment (arrow), consistent with vascular Eagle syndrome (stylocarotid syndrome) as a contributing mechanical factor.

Routine blood tests were unremarkable, including inflammatory markers (CRP <1 mg/L). Non-contrast CT brain demonstrated no acute intracranial abnormality. CT angiography (CTA) of the head and neck demonstrated left ICA dissection, with progressive luminal narrowing from the common carotid bifurcation to near-complete occlusion at the carotid foramen (Figure 1A, arrow; Figure 1B, arrow). Bone window and reconstructed views identified an elongated styloid process in close proximity to the affected carotid segment (Figure 1C, arrow), supporting vascular Eagle syndrome (stylocarotid syndrome) as a contributing mechanical factor. MRI brain demonstrated no diffusion restriction and no acute infarction.

Treatment

The patient was treated acutely with antiplatelet therapy with aspirin. Following imaging confirmation of carotid dissection without evidence of acute infarction, antiplatelet management was continued by transitioning to clopidogrel (secondary prevention), with longer-term planning and follow-up imaging surveillance. Repeat CTA was planned at interval follow-up to reassess the dissection and guide further management decisions. In view of the suspected stylocarotid mechanism, specialist multidisciplinary review was arranged to consider whether styloidectomy or vascular surgical input might be required to reduce the risk of recurrence.

Outcome

The patient's headache settled with regular simple analgesia. He remained neurologically intact throughout admission. MRI brain confirmed no acute infarction. He was discharged on antiplatelet therapy with stroke specialist follow-up. Interval CTA at six months was planned to monitor dissection healing and guide ongoing management, including review for potential definitive management of vascular Eagle syndrome depending on follow-up imaging and multidisciplinary assessment.

## Discussion

ICA dissection is a clinically important cause of stroke in young adults. Although it can follow trauma or be associated with connective tissue disorders, many cases are idiopathic. Mechanical irritation from an elongated styloid process is an under-recognised contributor, termed vascular Eagle syndrome or stylocarotid syndrome.

This case reinforces several key diagnostic lessons. First, severe new headache with atypical features warrants consideration of cervical artery dissection even when early non-contrast CT brain imaging is normal; identification of carotid dissection on CTA in this case precluded unnecessary lumbar puncture. Second, CTA is valuable not only for diagnosing dissection but also for identifying anatomical risk factors such as styloid elongation. Third, recognition of stylocarotid syndrome carries direct management implications, as recurrence may occur unless the mechanical trigger is addressed [1]. A limitation of this case is the absence of precise styloid process measurements and quantified ICA-to-styloid distance, which would provide stronger anatomical evidence for the stylocarotid mechanism.

The styloid process, first described by Eagle in 1937, lies in close proximity to the ICA and typically measures 2.5-3 cm [2]. Elongation beyond 3 cm or unfavourable angulation can lead to repetitive arterial irritation, particularly during physiological neck movement, resulting in endothelial injury, dissection, and thromboembolic complications [3,4]. Patients may present with unilateral neck pain, headache, Horner syndrome, transient ischaemic attacks, or stroke; however, presentation may be subtle, and a normal neurological examination does not exclude dissection.

Similar cases have been described in the literature, with presentations ranging from isolated pain syndromes to transient neurological deficits and established stroke [1,3-9]. Management has varied from conservative antiplatelet or anticoagulation therapy to endovascular stenting and/or styloidectomy, particularly where symptoms recurred, bilateral disease was also present, or ongoing mechanical irritation conferred high risk [1,6,8]. A recent systematic review demonstrated that patients who underwent styloidectomy were three times as likely to experience complete symptom resolution and 18 times less likely to experience symptom recurrence [1]. These reports support the importance of multidisciplinary input, typically involving stroke medicine, neuroradiology, ENT/maxillofacial surgery, and vascular teams, to individualise antithrombotic strategy, imaging surveillance, and consideration of definitive surgical intervention in selected patients.

## Conclusions

Vascular Eagle syndrome (stylocarotid syndrome) is an under-recognised but potentially reversible mechanical risk factor for ICA dissection. CTA is the key diagnostic modality, enabling both diagnosis of dissection and identification of structural contributors. Clinicians should maintain a low threshold for vascular imaging in young patients with severe new-onset headache, even in the absence of objective neurological deficit, and should consider multidisciplinary review where stylocarotid syndrome is identified.
